# Triple versus LAMA/LABA combination therapy for patients with COPD: a systematic review and meta-analysis

**DOI:** 10.1186/s12931-021-01777-x

**Published:** 2021-06-22

**Authors:** Akira Koarai, Mitsuhiro Yamada, Tomohiro Ichikawa, Naoya Fujino, Tomotaka Kawayama, Hisatoshi Sugiura

**Affiliations:** 1 Department of Respiratory Medicine, Tohoku University Graduate School of Medicine, 1-1 Seiryo-machi, Aoba-ku, Sendai, 980-8574 Japan; 2 Division of Respirology, Neurology and Rheumatology, Department of Medicine, Kurume University School of Medicine, 67 Asahi-machi, Kurume, 830-0011 Japan

**Keywords:** Chronic obstructive pulmonary disease, Exacerbations, Inhaled corticosteroid, Mortality, Pneumonia

## Abstract

**Background:**

Recently, the addition of inhaled corticosteroid (ICS) to long-acting muscarinic antagonist (LAMA) and long-acting beta-agonist (LABA) combination therapy has been recommended for patients with COPD who have severe symptoms and a history of exacerbations because it reduces the exacerbations. In addition, a reducing effect on mortality has been shown by this treatment. However, the evidence is mainly based on one large randomized controlled trial IMPACT study, and it remains unclear whether the ICS add-on treatment is beneficial or not. Recently, a large new ETHOS trial has been performed to clarify the ICS add-on effects. Therefore, we conducted a systematic review and meta-analysis to evaluate the efficacy and safety including ETHOS trial.

**Methods:**

We searched relevant randomized control trials (RCTs) and analyzed the exacerbations, quality of life (QOL), dyspnea symptom, lung function and adverse events including pneumonia and mortality, as the outcomes of interest.

**Results:**

We identified a total of 6 RCTs in ICS add-on protocol (N = 13,579). ICS/LAMA/LABA treatment (triple therapy) significantly decreased the incidence of exacerbations (rate ratio 0.73, 95% CI 0.64–0.83) and improved the QOL score and trough FEV_1_ compared to LAMA/LABA. In addition, triple therapy significantly improved the dyspnea score (mean difference 0.33, 95% CI 0.18–0.48) and mortality (odds ratio 0.66, 95% CI 0.50–0.87). However, triple therapy showed a significantly higher incidence of pneumonia (odds ratio 1.52, 95% CI 1.16–2.00). In the ICS-withdrawal protocol including 2 RCTs, triple therapy also showed a significantly better QOL score and higher trough FEV_1_ than LAMA/LABA. Concerning the trough FEV_1_, QOL score and dyspnea score in both protocols, the differences were less than the minimal clinically important difference.

**Conclusion:**

Triple therapy causes a higher incidence of pneumonia but is a more preferable treatment than LAMA/LABA due to the lower incidence of exacerbations, higher trough FEV_1_ and better QOL score. In addition, triple therapy is also superior to LABA/LAMA due to the lower mortality and better dyspnea score. However, these results should be only applied to patients with symptomatic moderate to severe COPD and a history of exacerbations.

*Clinical Trial Registration:* PROSPERO; CRD42020191978.

**Supplementary Information:**

The online version contains supplementary material available at 10.1186/s12931-021-01777-x.

## Background

Chronic obstructive pulmonary disease (COPD) is the third leading cause of death in the world [1]. The symptoms include dyspnea, cough and sputum production and worsen during exacerbations of COPD, which are associated with accelerated mortality [2]. To reduce the symptoms and the exacerbation, single or dual inhaled bronchodilators are recommended for the treatment depending on the severity. If the patients have severe symptoms and a history of exacerbations, the addition of inhaled corticosteroid (ICS) to long-acting muscarinic antagonist (LAMA) and long-acting beta-agonist (LABA) combination therapy has been recommended because it lowers the incidence of exacerbations [3]. Recently, a reduction of the mortality has also been shown by this treatment [4]. However, in severe COPD patients, the additional treatment of ICS could increase the incidence of pneumonia [3, 5, 6]. Therefore, a decision for the long-term use of ICS should be based on the total benefit for such patients.

Until now, five systematic reviews have been performed to evaluate the efficacy and safety of ICS add-on to LAMA/LABA treatment [7–11]. However, the included trials contain several biases, such as not using a single inhalation device [12], using different LABA between the comparison groups [13] or a short evaluation duration of only 24 weeks [14]. Therefore, the evidence from these systematic reviews is mainly from one large randomized controlled trial (RCT), the IMPACT study which was performed for 52 weeks using a single inhaler device [4], and it remains unclear whether the ICS add-on treatment is beneficial or not. Recently, a large new ETHOS trial has been performed to clarify the ICS add-on effects [15]. Therefore, we conducted a systematic review and meta-analysis to evaluate the efficacy and safety including ETHOS trial.

We searched relevant randomized control clinical trials and evaluated the efficacy and safety of ICS/LAMA/LABA (triple) versus LAMA/LABA therapy by measuring exacerbations, quality of life (QOL), dyspnea score, lung function and adverse events including pneumonia and mortality. We also compared the results of a meta-analysis of ICS add-on to LAMA/LABA (ICS add-on) with those in ICS withdrawal from triple therapy (ICS withdrawal).

## Methods

### Search strategy and eligibility criteria

This systematic review and meta-analysis was conducted according to the Preferred Reporting Items for Systematic Reviews and Meta-Analyses (PRISMA) guidance [16]. The study protocol was registered in the PROSPERO database (www.crd.york.ac.uk/prospero/; registration number: CRD42020191978). We first set outcomes based on the clinical importance and then performed a systematic literature review. We searched and identified RCTs in MEDLINE and the Cochrane Central Register of Controlled Trials (CENTRAL) including PubMed, EMBASE databases and ClinicalTrials.gov in June 2020, using the search strategy provided in the on-line supplement [17]. Only publications in English were considered. As the inclusion criteria, participants had a diagnosis of COPD according to the GOLD report’s diagnostic criteria. Randomized controlled trials comparing triple with LAMA/LABA therapy were included if they evaluated any of our outcomes of interest for a treatment duration at least 12 weeks. Unblinded or cross-over studies were excluded from the analysis because of the unblinded bias or the short treatment duration.

### Data collection and risk of bias assessment

At least two review authors (AK, MY, TI and NF) screened the titles and abstracts of all studies identified by the search strategy to check their eligibility. Next, full text assessments were performed to identify the studies for inclusion, and the data were retrieved from among the eligible studies. At least two review authors (AK, MY, TI and NF) assessed the risk of bias in the eligible studies according to the recommendations in the Cochrane Handbook for Systematic Reviews of Interventions 5.1.0. If there were discrepancies in the data collection or assessment of the risk of bias, the review authors resolved the disagreements through a discussion.

### Outcomes of interest

The included outcomes of interest in the current study were as follow: (i) exacerbations (number of patients experiencing one or more exacerbations per year or person-year), (ii) St George’s Respiratory Questionnaire (SGRQ) score change from the baseline, (iii) transitional dyspnea index (TDI) score change from the baseline, (iv) trough forced expiratory volume in one second (FEV_1_) change from the baseline, and (v) adverse events (total adverse events, serious adverse events, and pneumonia and mortality).

### Statistical analysis

We analyzed the data for the exacerbations as the rate ratio, dichotomous data as Mantel–Haenzsel odds ratios (ORs) and continuous data as mean difference with 95% confidence intervals (CIs) using the inverse variance (IV) test. Data were analyzed using Review Manager Software version 5.3 (Cochrane Library Software, Oxford, UK). We carefully checked whether the data were shown with standard deviation in each study and analyzed the data after conversion from standard error to standard deviation if the data were shown as standard error. Inconsistencies among the studies were assessed by the I^2^ statistic test. Publication bias was examined using funnel plots and assessed visually when applicable. Subgroup analyses were performed in the cause of mortality, the background of participants who had a history of exacerbations in previous year and ≥ 10 COPD Assessment Test (CAT) score and the blood eosinophil level. The quality of evidence was measured according to the Grading of Recommendations Assessment, Development and Evaluation (GRADE) system, and absolute estimates of the effect for the outcomes were also evaluated [18].

## Results

### Characteristics of selected studies

**Fig. 1:**
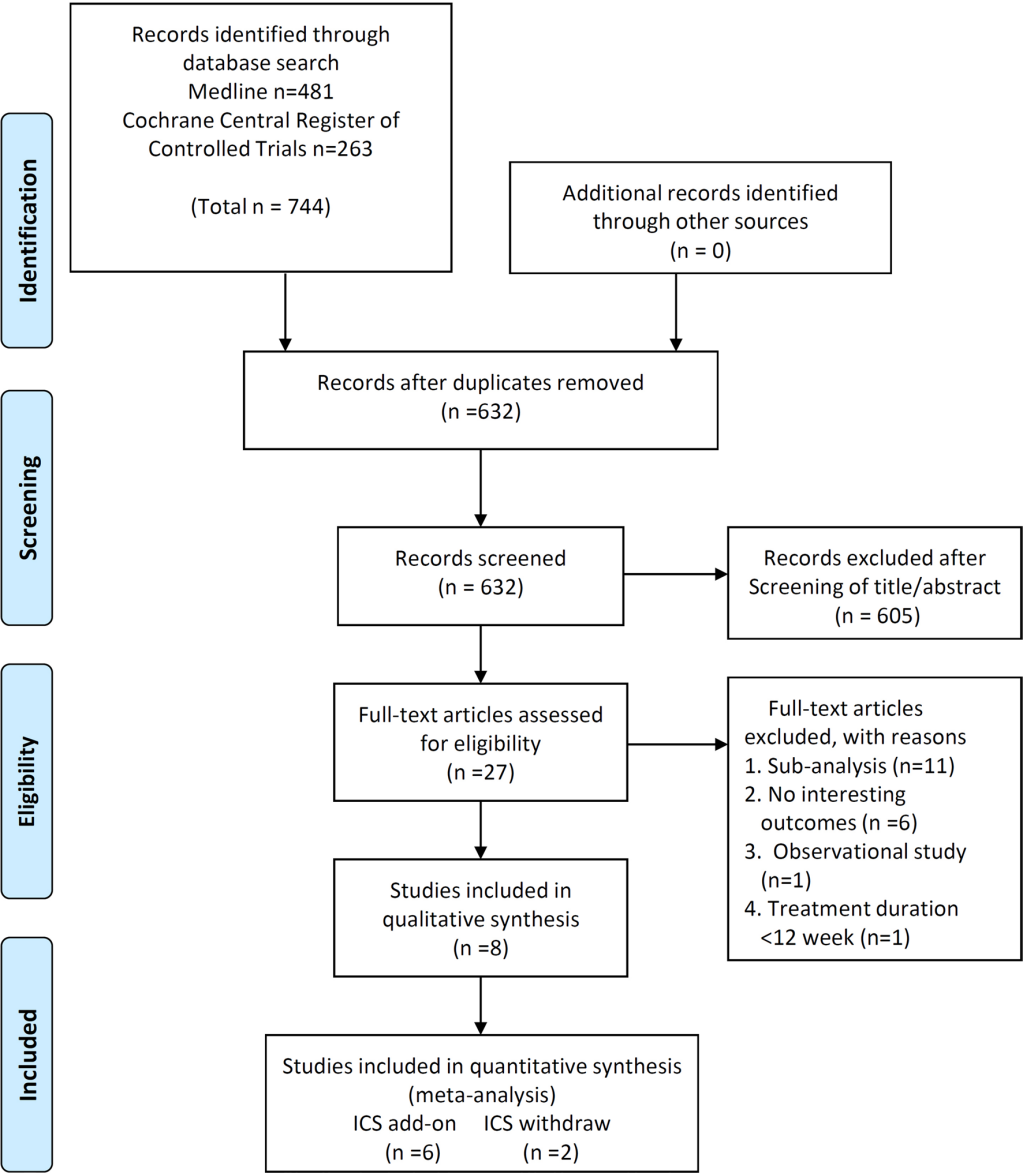
Flow diagram of study selection

The search strategy yielded 632 candidate studies, excluding duplicates. After full-text assessment, we excluded 19 trials and finally identified a total of 6 RCTs eligible for the meta-analysis in the ICS add-on protocol (N = 13,579) and a total of 2 RCTs (N = 3538) in the ICS withdrawal protocol (Fig. 1 and Additional file 1: Table S1). These studies were published from 2002 to 2020 and their characteristics are summarized in Table 1 and in the Additional file 1: Table S2, S3. The participants were at least 35 years of age, current or ex-smokers with a smoking history of 10 pack-years or more, and the severity of the disease was moderate to severe. The treatment period was 24 to 52 weeks. Concerning the history of exacerbations, six studies [4, 12, 13, 15, 19, 20] required a history of exacerbations in previous years, but two studies did not [14, 21]. Patients with current asthma were excluded in all eight studies, but a previous history of asthma was not excluded except in one study [20]. In the ICS add-on protocol, 65 – 80% of those in the trial’s population were taking ICS at screening (Additional file 1: Table S2).

**Table 1:**
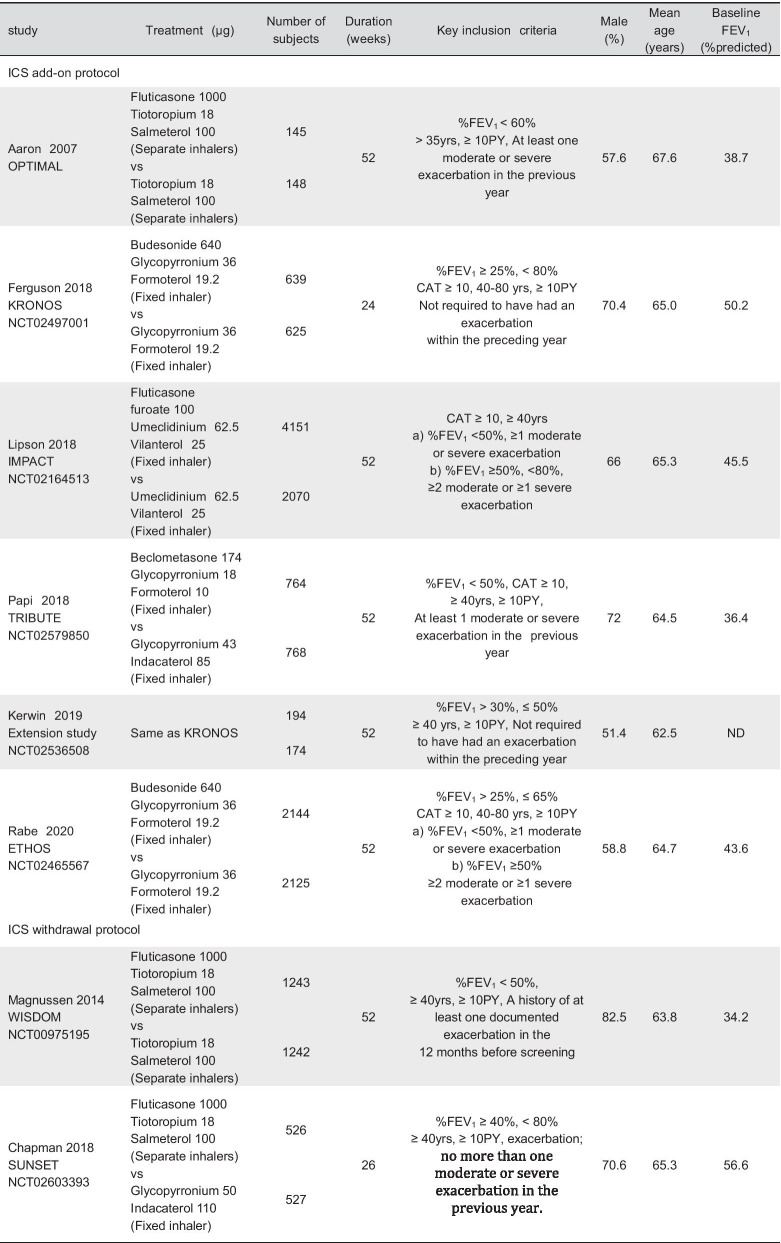
Characteristics of included studies

Moderate exacerbation is defined as requiring antibiotics and/or oral steroids, and severe exacerbation is defined as requiring hospitalization

*FEV*_*1*_  forced expiratory volume in 1 s, *yrs* years, *PY*  pack-years, *CAT*  COPD Assessment Test, *ND*  not demonstrated

### Risk of bias

The risks of selection bias and performance bias were low. Unclear risk in the blinding of outcome assessment was found in four studies. Three trials had an unclear risk in the incomplete outcome data. In other biases, seven studies were contained unclear risk because the sponsors were all pharmaceutical companies (Additional file 1: Tables S4, S5). Concerning publication bias, funnel plots were not suitable for the assessment because they cannot be interpreted accurately if the number of studies is less than 10 [22]. Therefore, publication bias was assessed with our comprehensive on-line database searches and considered not to be seen.

### Outcome assessments

#### Exacerbations

**Fig. 2:**
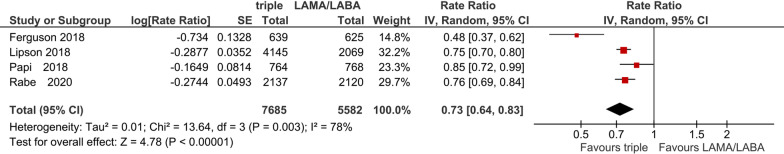
Efficacy of ICS add-on to LAMA/LABA on exacerbations

Four studies with 13,267 participants were included for the evaluation of exacerbations in the ICS add-on protocol. There was a significant decrease in the incidence of exacerbations with ICS/LAMA/LABA when compared with LAMA/LABA (rate ratio 0.73, 95% CI 0.64 to 0.83; P < 0.00001; I^2^ = 78%; Fig. 2 and Additional file 1: Figure S1).

#### SGRQ score

**Fig. 3:**
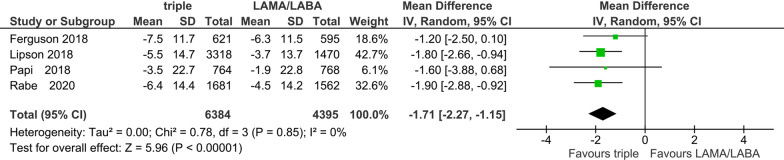
Efficacy of ICS add-on to LAMA/LABA on quality of life: change from baseline in SGRQ

Four studies with 10,779 participants were included for the evaluation of the SGRQ score in the ICS add-on protocol. There was a significant improvement in the SGRQ score change from the baseline with ICS/LAMA/LABA (mean difference  − 1.71, 95% CI − 2.27 to − 0.92; P < 0.00001; I^2^ = 0%; Fig. 3). However, this difference was less than the minimal clinically important difference (MCID) of − 4.0 [23, 24].

#### TDI score

**Fig. 4:**
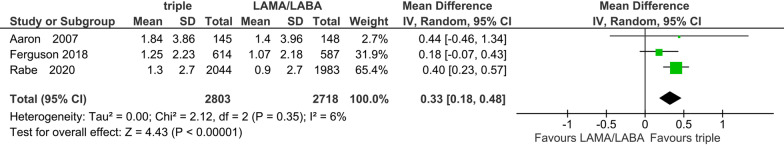
Efficacy of ICS add-on to LAMA/LABA on symptoms: change from baseline in TDI score

Three studies with 5521 participants were included for the evaluation of the TDI score in the ICS add-on protocol. There was a significant improvement in the TDI score change from the baseline with ICS/LAMA/LABA (mean difference 0.33, 95% CI 0.18 to 0.48; P < 0.00001; I^2^ = 6%; Fig. 4). However, this difference was less than the MCID of 1.0 [23, 24].

#### Trough FEV_1_

**Fig. 5:**
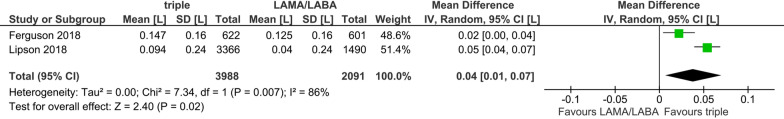
Efficacy of ICS add-on to LAMA/LABA on trough FEV_1_: change from baseline

Two studies with 6079 participants were included for the evaluation of the trough FEV_1_ in the ICS add-on protocol. Compared to LAMA/LABA, there was a significant increase in the trough FEV_1_ with ICS/LAMA/LABA (mean difference 0.04, 95% CI 0.01 to 0.07, P = 0.02, I^2^ = 86%; Fig. 5). However, this difference was less than the MCID of 0.05 to 0.10 L [23–25].

#### Adverse events

Five studies with 12,683 participants were included for the evaluation of adverse events in the ICS add-on protocol. There was no difference in the total adverse events between ICS/LAMA/LABA and LAMA/LABA (OR 1.03, 95% CI 0.93 to 1.15; P = 0.58; I^2^ = 34%; Additional file 1: Figure S2). In the serious adverse events, there was also no difference between them (OR 0.95, 95% CI 0.87 to 1.04; P = 0.28; I^2^ = 0%; Additional file 1: Figure S3).

#### Pneumonia events

**Fig. 6:**
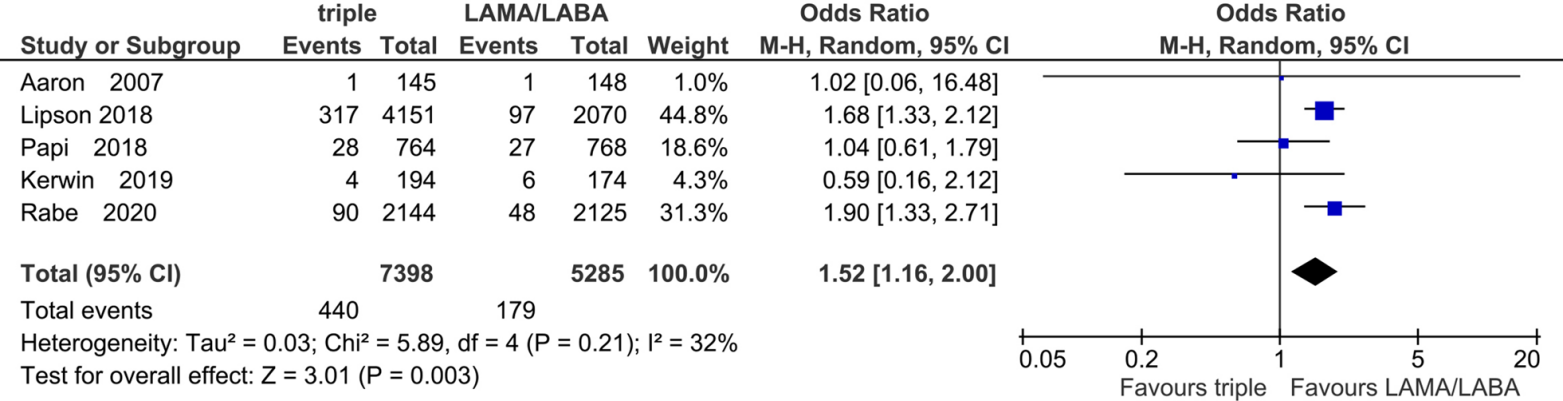
Efficacy of ICS add-on to LAMA/LABA on pneumonia events

Five studies with 12,683 participants were included for the evaluation of pneumonia events in the ICS add-on protocol. The treatment periods in the five studies were all 52 weeks. Compared to LAMA/LABA, there was a significant increase in the pneumonia events with ICS/LAMA/LABA (OR 1.52, 95% CI 1.16 to 2.00; P = 0.003; I^2^ = 32%; Fig. 6).

#### Mortality

**Fig. 7:**
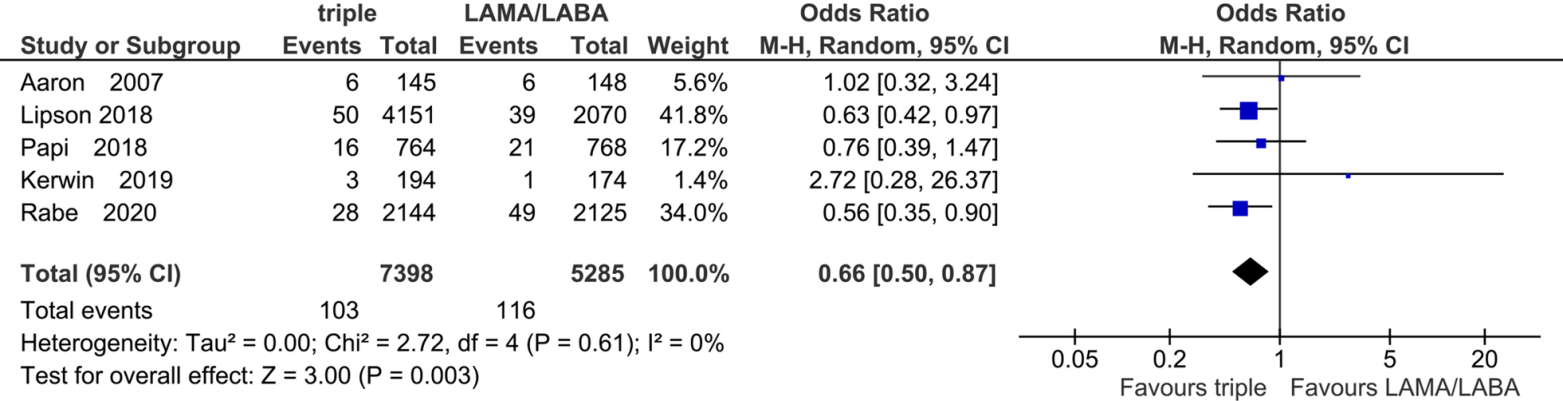
Efficacy of ICS add-on to LAMA/LABA on mortality

Five studies with 12,683 participants were included for the evaluation of mortality events in the ICS add-on protocol. The treatment periods in the five studies were all 52 weeks. The median incidence was 1.6% in ICS/LAMA/LABA and 2.3% in LAMA/LABA. The incidence was small, but the frequency with ICS/LAMA/LABA was significantly lower (OR 0.66, 95% CI 0.50 to 0.87; P = 0.003; I^2^ = 0%; Fig. 7). In the sub-analysis of cause of mortality, fatal cardiovascular events with ICS/LAMA/LABA were significantly lower than those with LAMA/LABA (OR 0.50, 95% CI 0.31 to 0.80; P = 0.004; I^2^ = 0%; Additional file 1: Figure S4).

#### Sub-analysis with history of exacerbations and CAT score

Sub-analysis was performed in the participants who had a history of exacerbations in the previous year and ≥ 10 CAT score, which is mainly included in the GOLD group D [3] (Additional file 1: Figure S5 – S10). When compared with LAMA/LABA, the sub-analysis showed a significantly lower incidence of exacerbations (rate ratio 0.76, 95% CI 0.72 to 0.80; P < 0.00001; I^2^ = 0%; Additional file 1: Figure S5) and mortality (rate ratio 0.63, 95% CI 0.47 to 0.83; P = 0.001; I^2^ = 0%; Additional file 1: Figure S10), and better SGRQ score with ICS/LAMA/LABA (mean difference − 1.83, 95% CI − 2.45 to − 1.20; P < 0.00001; I^2^ = 0%; Additional file 1: Figure S6). However, there was a significantly higher incidence of pneumonia with ICS/LAMA/LABA treatment (rate ratio 1.60, 95% CI 1.23 to 2.09; P = 0.0005; I^2^ = 41%; Additional file 1: Figure S9). Concerning the TDL score and trough FEV_1_, data from one trial showed a significant improvement in the TDL score (mean difference 0.40, 95% CI 0.23 to 0.57; P < 0.00001; I^2^: not applicable; Additional file 1: Figure S7) and trough FEV_1_ with ICS/LAMA/LABA (mean difference 0.05, 95% CI 0.04 to 0.07, P < 0.00001, I^2^: not applicable; Additional file 1: Figure S8).

#### Comparison between ICS add-on and ICS withdrawal protocol

There were two trials (N = 3538) evaluating the effects of ICS withdrawal from ICS/LAMA/LABA. Compared to the ICS add-on protocol, there were no differences between ICS/LAMA/LABA and LAMA/LABA in the rate of exacerbations, TDI score, and total and serious adverse events including pneumonia and mortality (Additional file 1: Figures S1, and S11–S18). However, triple therapy showed a significant better SGRQ score (mean difference − 1.33 95% CI − 2.26 to − 0.40; P = 0.005; I^2^ = 0%) and higher trough FEV_1_ (mean difference 0.04, 95% CI 0.02 to 0.05, P = 0.0005, I^2^ = 0%) same as in ICS add-on protocol (Additional file 1: Figures S12 and S14).

#### Sub-analysis with baseline blood eosinophil count (descriptive analysis)

There were four trials that evaluated the ICS add-on effect and one trial that assessed the ICS withdrawal effect based on the baseline blood eosinophil count during exacerbations (Additional file 1: Table S6). All the studies have shown that the patients with higher level of eosinophils, such as ≥ 150 or 300, experienced markedly greater reductions in moderate or severe COPD exacerbations. Also, each trial evaluated the ICS add-on or ICS withdrawal effect based on the eosinophils in the trough FEV_1_ and showed a higher level of trough FEV_1_ if the level of eosinophils was greater than ≥ 150 or 300 (Additional file 1: Table S7).

#### Evaluation with GRADE

The overall quality of evidence was high for outcomes including exacerbations, SGRQ score, total adverse events, serious adverse events and pneumonia, and was moderate for the TDI score, trough FEV_1_ and mortality (Additional file 1: Table S8). When ICS were added to LAMA/LABA for the 1,000 patients and the rate of exacerbation with LAMA/LABA was assumed to be 1.0 exacerbation event per person-year, 270 (95%CI 360 to 170) fewer exacerbations, 7 (95% CI 3 to 11) fewer mortalities and 17 (95%CI 5 to 32) more pneumonia events would have been experienced.

## Discussion

In the current meta-analysis, we first evaluated the efficacy and safety in the comparison between ICS/LAMA/LABA and LAMA/LABA for the patients with COPD including those in the ETHOS trial. In the patients with symptomatic moderate and severe COPD and a history of exacerbations, we demonstrated that the addition of ICS to LABA/LAMA caused a higher incidence of pneumonia than LAMA/LABA but was a more preferable treatment due to the lower incidence of exacerbations, higher trough FEV_1_ and better QOL score. We also revealed that triple therapy was superior to LAMA/LABA due to the lower mortality and better dyspnea score in these patients.

Until now, five systematic reviews have compared the effect of triple therapy with LAMA/LABA in patients with COPD [7–11]. These reviews showed that triple therapy has a risk of pneumonia but is superior to LAMA/LABA therapy due to the lower incidence of exacerbations, higher trough FEV_1_ and better QOL score evaluated by GRADE. In the safety components, these reviews also showed that there were no differences in the total and serious adverse events between triple and LAMA/LABA therapy. However, these results were mainly based on the data from one large IMPACT study [4]. In the current meta-analysis, we confirmed these results by using additional data from the ETHOS trial [15]. In addition, we demonstrated for the first time that triple therapy was superior to LAMA/LABA therapy as reflected by the lower mortality and better dyspnea score. This difference could be mainly due to the increase of participants together with the inclusion of one large ETHOS study [15]. Also, we evaluated the effect of ICS add-on to LAMA/LABA on the mortality and pneumonia events in COPD patients with a 52 week treatment duration. However, around 80% of the participants for most variables were composed of those from the IMPACT and ETHOS trials [4, 15], which aimed at evaluating exacerbations in the participants with ≥ 10 CAT score and a history of exacerbations. Therefore, attention should be given when the results applied.

Concerning exacerbations, the four trials evaluated all demonstrated the significant superiority of triple therapy to LAMA/LABA in reducing the risk of exacerbations. However, there was a high grade of inconsistency in the meta-analysis. This might be due to differences in the inclusion criteria for the participants with a history of exacerbations in the previous year. Three trials included a history of ≥ 1 moderate or severe exacerbations for the inclusion criteria, but one KRONOS trial excluded. When the KRONOS trial was excluded in the sub-analysis, the inconsistency disappeared. The KRONOS trial that mainly included patients with a history of less than one moderate or severe exacerbation in the previous year, also showed the superiority of triple therapy to LAMA/LABA in reducing the risk of exacerbations. However, there have been insufficient trials that examined patients with a history of less than one exacerbation to confirm this result.

In the trough FEV_1_, we confirmed the superiority of triple therapy to LAMA/LABA. The difference of 40 mL is below the MCID of 50–100 mL, for which the index is usually used for comparison with a placebo [23–25]. However, in our present analysis, the difference in the trough FEV_1_ might have caused a significant change in the patient’s QOL and dyspnea symptoms evaluated by the SGRQ and TDI score and a decrease of exacerbations because relationships between improvement in FEV_1_ and QOL or exacerbations in COPD have been reported [26, 27]. In addition, a stronger relationship between the improvement in FEV_1_ and QOL has been shown in more severe COPD [26]. Therefore, the degree of change in trough FEV_1_ in the current analysis may affect the clinical course in the patients with more severe COPD.

Concerning the mortality, our meta-analysis demonstrated that triple therapy was associated with a significantly lower mortality in patients with COPD compared with LAMA/LABA. This result is consistent with previous studies that suggested ICS/LABA or ICS/LAMA/LABA causes a reduction in mortality in patients with COPD [28–32]. The effect of ICS on the mortality might be dose-dependent because a half dose of ICS treatment with LAMA/LABA did not reduced the mortality in the ETHOS trial [15]. In the sub-analysis of cause of the mortality, we showed that the reduction of mortality with triple therapy might be mainly due to a lower rate of fatal cardiovascular events. However, the results of a recent SUMMIT trial which aimed at evaluating the impact of ICS or ICS/LABA on the reduction of cardiovascular events and mortality, were negative. This discrepancy may be mainly due to differences in the severity and the history of exacerbations because the participants in the SUMMIT study suffered less severe COPD with a lower rate of exacerbations in the previous year [30]. Previous studies have shown that exacerbations of COPD could increase the risk of coronary and stroke events [33, 34]. Therefore, the protective effect of triple therapy on exacerbations could lead to a lower incidence of fatal cardiac events. However, there was an imprecision in our current results because the sample size was statistically still not sufficient and the estimated duration of less than 52 weeks also not long enough for evaluating mortality. Further trials are awaited to confirm these results.

In the sub-analysis that evaluated the patients with ≥ 10 CAT score and a history of ≥ 1 moderate or severe exacerbations in the previous year, the criteria of that covers GOLD group D, triple therapy showed a significantly lower incidence of exacerbations and mortality, and improvement in the SGRQ score, but a higher incidence of pneumonia. Concerning the TDL score and trough FEV_1_, the superiority of triple therapy was also shown in the sub-analysis evaluated in one study. Therefore, the patients in GOLD group D could be the main targets for ICS add-on to LAMA/LABA.

In the ICS withdrawal protocol, there was no significant difference between triple and LAMA/LABA therapy in the incidence of exacerbations and pneumonia. These results are inconsistent with those from the ICS add-on protocol. This inconsistency might be mainly explained by differences in the protocol and the basal control level of the COPD status, such as the exacerbation rate, which has been shown to be a risk for future exacerbations and pneumonia [35, 36]. In fact, in the ICS withdrawal protocol, the participants in the SUNSET trial experienced no more than one moderate or severe exacerbation in the previous year. Also, in the WISDOM trial, the participants could also have been less frequent exacerbators because the exacerbation rate in the LAMA/LABA group was lower than those in the two large IMPACT and ETHOS trials in the ICS add-on protocol (Additional file 1: Figure S1). On the other hand, our analysis demonstrated that triple therapy showed a significantly better SGRQ score and higher trough FEV_1_ than LAMA/LABA in both protocols. This result suggests that, although the degree of improvement in the SGRQ score and trough FEV_1_ with triple therapy was less than MCID, the certainty of this evidence is quite high.

In our current analysis, we did not address what factors determined the effectiveness of triple therapy because detailed data were not reported in the included trials except blood eosinophil count. A higher number of blood eosinophils has been shown to reflect the eosinophilic airway inflammation, which is a steroid sensitive element in asthmatic patients [37, 38]. Our current analysis and the post hoc analyses have shown a correlation between blood eosinophil counts and the efficacy [39, 40]. However, since various cut-off values, such as ≥ 150 or 300 of blood eosinophils were used in each trial, the most useful value remains unclear. In our current meta-analysis, all studies excluded current asthma but not the patients with a previous history of asthma except one, the SUNSET trial [20]. Also, around 15 to 20% of patients were included with ≥ 300 blood eosinophil counts at the baseline (Additional file 1: Table S3) [40], suggesting that these trials could have contained a selection bias that includes asthma patients potentially responsive to ICS. The previous FLAME trial, which excluded patients with current or previous asthma history, reported that the LAMA/LABA treatment showed a lower incidence of exacerbations of COPD than ICS/LABA [41]. Therefore, "pure COPD" patients might be less responsive to ICS, but further studies are needed to clarify this point.

There remains a possibility that prior ICS usage might also have affected the ICS add-on effect. In our current analysis, 65 – 80% of the trial’s population were taking ICS at screening and without any washing period except the TRIBUTE study before starting the trial in the ICS add-on protocol. Therefore, these trials cannot be strictly defined as an ICS add-on. In a post-hoc analysis of the IMPACT trial, the ICS add-on effect on the moderate or severe exacerbations was reduced among prior ICS nonusers, but not in that of the ETHOS trial [15, 42]. On the other hand, both trials have shown no apparent difference in the mortality in the ICS nonusers with a limited sample size [43, 44]. Therefore, the results in the ICS nonusers remain unclear but the impact of ICS withdrawal could have been anticipated to be small because these trials demonstrated that both therapeutic and adverse effects could last after three months when excluding the influence of ICS withdrawal [42–44].

There are several limitations to our meta-analysis. First, the included patients had less than 80% of %FEV_1_; therefore, current results are not applicable to mild COPD patients. Secondly, the participants included in our meta-analysis in the ICS add-on protocol were mainly limited to those with history of smoking, CAT score of ≥ 10 and a history of exacerbations in the previous year and without current asthma. Thirdly, the inconsistency of some meta-analyses might be due to differences in drugs that compose ICS/LAMA/LABA. However, we did not evaluate the difference of drugs in the sub-group analysis because of the lack of trials.

## Conclusions

In the patients with symptomatic moderate and severe COPD and a history of exacerbations, triple therapy causes a higher incidence of pneumonia than LAMA/LABA, but is still a more preferable treatment due to the lower incidence of exacerbations, higher trough FEV_1_ and better QOL score. In these patients, triple therapy was also superior to LAMA/LABA due to the lower mortality and better dyspnea score.

## Supplementary Information

Additional file 1: **Table S1.** List of studies excluded from the analysis. **Table S2.** Characteristics of included studies for the analysis of each outcome. **Table S3.** Baseline blood eosinophil count and moderate to severe COPD exacerbations in the past 12 months. **Table S4.** Assessment of risk of bias. **Table S5.** Details for the risk bias assessment. **Table S6.** Sub-analysis of exacerbations by baseline blood eosinophil count (descriptive analysis). **Table S7.** Sub-analysis of trough FEV_1_ by baseline blood eosinophil count (descriptive analysis). **Table S8.** Summary of findings for the main comparison. **Figure S1.** Comparison of exacerbation rate in each trial. N.S. = not significant; N.A. = not available from the original papers. **Figure S2.** Efficacy of ICS add-on to LAMA/LABA on total adverse events. **Figure S3.** Efficacy of ICS add-on to LAMA/LABA on serious adverse events. **Figure S4.** Sub-analysis of cause of mortality: cardiovascular events. **Figure S5.** Sub-analysis of exacerbations by history of exacerbations and CAT score. **Figure S6.** Sub-analysis of SGRQ score by history of exacerbations and CAT score. **Figure S7.** Sub-analysis of TDI sore by history of exacerbations and CAT score. **Figure S8.** Sub-analysis of trough FEV_1_ by history of exacerbations and CAT score. **Figure S9.** Sub-analysis of pneumonia events by history of exacerbations and CAT score. **Figure S10.** Sub-analysis of mortality by history of exacerbations and CAT score. **Figure S11.** Efficacy of ICS withdrawal from ICS/LAMA/LABA on exacerbations. **Figure S12.** Comparison between ICS add-on and ICS withdrawal protocol: change from baseline in SGRQ score. **Figure S13.** Comparison between ICS add-on and ICS withdrawal protocol: change from baseline in TDI score. **Figure S14.** Comparison between ICS add-on and ICS withdrawal protocol: trough FEV_1_. **Figure S15.** Comparison between ICS add-on and ICS withdrawal protocol: total adverse events. **Figure S16.** Comparison between ICS add-on and ICS withdrawal protocol: serious adverse events. **Figure S17.** Comparison between ICS add-on and ICS withdrawal protocol: pneumonia events. **Figure S18.** Comparison between ICS add-on and ICS withdrawal protocol: mortality.

## Data Availability

Source data and material will be made available upon reasonable request.

## Abbreviations

CAT: COPD Assessment Test
CENTRAL: Cochrane Central Register of Controlled Trials
CI: Confidence intervals
COPD: Chronic obstructive pulmonary disease
FEV_1_: Forced expiratory volume in one second
GOLD: Global initiative for chronic obstructive lung disease
LABAs: Long-acting beta-agonists
LAMAs: Long-acting muscarinic antagonist
MCID: Minimal clinically important difference
OR: Odds ratios
PRISMA: Preferred Reporting Items for Systematic Reviews and Meta-Analyses
SD: Standard deviation
SGRQ: St George’s Respiratory Questionnaire
TDI: Transitional dyspnea index
