# Supplementary material for: Triple versus LAMA/LABA combination therapy for patients with COPD: a systematic review and meta-analysis
Source: Respir Res. 2021 Jun 22;22:183. doi: 10.1186/s12931-021-01777-x (PMC8218448; doi:10.1186/s12931-021-01777-x)
Supplement: Supplementary file 1 — Additional file 1: Table S1. List of studies excluded from the analysis. Table S2. Characteristics of included studies for the analysis of each outcome. Table S3. Baseline blood eosinophil count and moderate to severe COPD exacerbations in the past 12 months. Table S4. Assessment of risk of bias. Table S5. Details for the risk bias assessment. Table S6. Sub-analysis of exacerbations by baseline blood eosinophil count (descriptive analysis). Table S7. Sub-analysis of trough FEV1 by baseline blood eosinophil count (descriptive analysis). Table S8. Summary of findings for the main comparison. Figure S1. Comparison of exacerbation rate in each trial. N.S. = not significant; N.A. = not available from the original papers. Figure S2. Efficacy of ICS add-on to LAMA/LABA on total adverse events. Figure S3. Efficacy of ICS add-on to LAMA/LABA on serious adverse events. Figure S4. Sub-analysis of cause of mortality: cardiovascular events. Figure S5. Sub-analysis of exacerbations by history of exacerbations and CAT score. Figure S6. Sub-analysis of SGRQ score by history of exacerbations and CAT score. Figure S7. Sub-analysis of TDI sore by history of exacerbations and CAT score. Figure S8. Sub-analysis of trough FEV1 by history of exacerbations and CAT score. Figure S9. Sub-analysis of pneumonia events by history of exacerbations and CAT score. Figure S10. Sub-analysis of mortality by history of exacerbations and CAT score. Figure S11. Efficacy of ICS withdrawal from ICS/LAMA/LABA on exacerbations. Figure S12. Comparison between ICS add-on and ICS withdrawal protocol: change from baseline in SGRQ score. Figure S13. Comparison between ICS add-on and ICS withdrawal protocol: change from baseline in TDI score. Figure S14. Comparison between ICS add-on and ICS withdrawal protocol: trough FEV1. Figure S15. Comparison between ICS add-on and ICS withdrawal protocol: total adverse events. Figure S16. Comparison between ICS add-on and ICS withdrawal protocol: serious adverse events. Fig [file 12931_2021_1777_MOESM1_ESM.docx]

**Triple versus LAMA/LABA combination therapy for patients with COPD: a systematic review and meta-analysis**

*Akira Koarai, M.D., Ph.D.^1^, Mitsuhiro Yamada, M.D., Ph.D.^1^, Tomohiro Ichikawa M.D., Ph.D.^1^, Naoya Fujino M.D., Ph.D.^1^, Tomotaka Kawayama, M.D., Ph.D. ^2^ and Hisatoshi Sugiura, M.D., Ph.D.^1^

^1^ Department of Respiratory Medicine, Tohoku University Graduate School of Medicine, 1-1 Seiryo-machi, Aoba-ku, Sendai 980-8574, Japan.

^2^ Division of Respirology, Neurology and Rheumatology, Department of Medicine, Kurume University School of Medicine, 67 Asahi-machi, Kurume 830-0011, Japan.

**Additional file 1**

**Search strategy for MEDLINE and CENTRAL**

| # | Searches |
| --- | --- |
| 1 | Lung Diseases, Obstructive/ |
| 2 | exp Pulmonary Disease, Chronic Obstructive/ |
| 3 | emphysema*.mp. |
| 4 | (chronic* adj3 bronchiti*).mp. |
| 5 | (chronic* adj3 (pulmonary or lung* or airway* or airfow* or bronch* or respirat*)).mp. |
| 6 | COPD.mp. |
| 7 | COAD.mp. |
| 8 | COBD.mp. |
| 9 | AECB.mp. |
| 10 | or/1-9 |
| 11 | clinical trial.pt. |
| 12 | (randomized or randomised).ab,ti. |
| 13 | placebo.ab,ti. |
| 14 | dt.fs. |
| 15 | randomly.ab,ti. |
| 16 | trial.ab,ti. |
| 17 | groups.ab,ti. |
| 18 | or/11-17 |
| 19 | Animals/ |
| 20 | Humans/ |
| 21 | 19 not (19 and 20) |
| 22 | 18 not 21 |
| 23 | Adrenergic beta-2 Receptor Agonists.sh. |
| 24 | (long* adj beta* adj agonist*).mp. |
| 25 | salmeterol*.mp. |
| 26 | formoterol*.mp. |
| 27 | indacaterol*.mp. |
| 28 | QAB-149.mp. |
| 29 | vilanterol*.mp. |
| 30 | GW642444.mp. |
| 31 | olodaterol*.mp. |
| 32 | BI 1744 CL.mp. |
| 33 | tulobuterol*.mp. |
| 34 | bambuterol*.mp. |
| 35 | clenbuterol*.mp. |
| 36 | or/23-35 |
| 37 | Muscarinic Antagonists.sh. |
| 38 | (muscarinic* adj antagonist*).mp. |
| 39 | LAMA.ab,ti. |
| 40 | tiotropium*.mp. |
| 41 | Spiriva.mp. |
| 42 | glycopyrronium*.mp. |
| 43 | glycopyrrolate*.mp. |
| 44 | NVA237.mp. |
| 45 | Seebri.mp. |
| 46 | umeclidinium*.mp. |
| 47 | GSK573719.mp. |
| 48 | Incruse.mp. |
| 49 | aclidinium*.mp. |
| 50 | LAS34273.mp. |
| 51 | Turdorza.mp. |
| 52 | Eklira.mp. |
| 53 | or/37-52 |
| 54 | Ultibro.mp. |
| 55 | QVA149.mp. |
| 56 | Stiolto.mp. |
| 57 | Anoro.mp. |
| 58 | Bevespi.mp. |
| 59 | or/54-58 |
| 60 | Adrenal Cortex Hormones Explode All.sh. |
| 61 | (inhal* adj (corticosteroid* or steroid* or glucocorticoid*)).mp. |
| 62 | fluticasone*.mp. |
| 63 | budesonide*.mp. |
| 64 | beclomethasone*.mp. |
| 65 | ciclesonide*.mp. |
| 66 | flunisolide*.mp. |
| 67 | mometasone*.mp. |
| 68 | triamcinolone*.mp. |
| 69 | Symbicort.mp. |
| 70 | Viani.mp. |
| 71 | Seretide.mp. |
| 72 | Advair.mp. |
| 73 | Atmadisc.mp. |
| 74 | Adoair.mp. |
| 75 | (Foster or Fostair).mp. |
| 76 | Inuvair.mp. |
| 77 | Dulera.mp. |
| 78 | Flutiform.mp. |
| 79 | Breo.mp. |
| 80 | Breztri.mp. |
| 81 | Trelegy.mp. |
| 82 | or/60-81 |
| 83 | 36 and 53 |
| 84 | 83 or 59 |
| 85 | 10 and 22 and 84 and 82 |

**Table S1** List of studies excluded from the analysis.

| **Study** | **Reason for exclusion** |
| --- | --- |
| Najafzadeh M, Marra CA, Sadatsafavi M, Aaron SD, Sullivan SD, Vandemheen KL, et al. Cost effectiveness of therapy with combinations of long acting bronchodilators and inhaled steroids for treatment of COPD. Thorax. 2008; 63:962-7. | No interesting outcomes |
| Bolukbas S, Eberlein M, Eckhoff J, Schirren J. Short-term effects of inhalative tiotropium/formoterol/budenoside versus tiotropium/formoterol in patients with newly diagnosed chronic obstructive pulmonary disease requiring surgery for lung cancer: a prospective randomized trial. Eur J Cardiothorac Surg. 2011; 39:995-1000. | Treatment duration <12 week |
| Rodriguez-Roisin R, Tetzlaff K, Watz H, Wouters EF, Disse B, Finnigan H, et al. Daily home-based spirometry during withdrawal of inhaled corticosteroid in severe to very severe chronic obstructive pulmonary disease. Int J Chron Obstruct Pulmon Dis. 2016; 11:1973-81. | No interesting outcomes |
| Watz H, Tetzlaff K, Wouters EF, Kirsten A, Magnussen H, Rodriguez-Roisin R, et al. Blood eosinophil count and exacerbations in severe chronic obstructive pulmonary disease after withdrawal of inhaled corticosteroids: a post-hoc analysis of the WISDOM trial. Lancet Respir Med. 2016; 4:390-8. | Sub-analysis |
| Buhl R, Criee CP, Kardos P, Vogelmeier CF, Kostikas K, Lossi NS, et al. Dual bronchodilation vs triple therapy in the "real-life" COPD DACCORD study. Int J Chron Obstruct Pulmon Dis. 2018; 13:2557-68. | Observational study |
| Watz H, Tetzlaff K, Magnussen H, Mueller A, Rodriguez-Roisin R, Wouters EFM, et al. Spirometric changes during exacerbations of COPD: a post hoc analysis of the WISDOM trial. Respir Res. 2018; 19:251. | Sub-analysis |
| Ichinose M, Fukushima Y, Inoue Y, Hataji O, Ferguson GT, Rabe KF, et al. Efficacy and Safety of Budesonide/Glycopyrrolate/Formoterol Fumarate Metered Dose Inhaler Formulated Using Co-Suspension Delivery Technology in Japanese Patients with COPD: A Subgroup Analysis of the KRONOS Study. Int J Chron Obstruct Pulmon Dis. 2019; 14:2979-91. | Sub-analysis |
| Ichinose M, Fukushima Y, Inoue Y, Hataji O, Ferguson GT, Rabe KF, et al. Long-Term Safety and Efficacy of Budesonide/Glycopyrrolate/Formoterol Fumarate Metered Dose Inhaler Formulated Using Co-Suspension Delivery Technology in Japanese Patients with COPD. Int J Chron Obstruct Pulmon Dis. 2019; 14:2993-3002. | Sub-analysis |
| Ismaila AS, Risebrough N, Schroeder M, Shah D, Martin A, Goodall EC, et al. Cost-Effectiveness Of Once-Daily Single-Inhaler Triple Therapy In COPD: The IMPACT Trial. Int J Chron Obstruct Pulmon Dis. 2019; 14:2681-95. | No interesting outcomes |
| Kato M, Tomii K, Hashimoto K, Nezu Y, Ishii T, Jones CE, et al. The IMPACT Study - Single Inhaler Triple Therapy (FF/UMEC/VI) Versus FF/VI And UMEC/VI In Patients With COPD: Efficacy And Safety In A Japanese Population. Int J Chron Obstruct Pulmon Dis. 2019; 14:2849-61. | Sub-analysis |
| Palli SR, Buikema AR, DuCharme M, Frazer M, Kaila S, Juday T. Costs, exacerbations and pneumonia after initiating combination tiotropium olodaterol versus triple therapy for chronic obstructive pulmonary disease. J. 2019; 8:1299-316. | No interesting outcomes |
| Pascoe S, Barnes N, Brusselle G, Compton C, Criner GJ, Dransfield MT, et al. Blood eosinophils and treatment response with triple and dual combination therapy in chronic obstructive pulmonary disease: analysis of the IMPACT trial. Lancet Respir Med. 2019; 7:745-56. | Sub-analysis |
| Singh D, Fabbri LM, Vezzoli S, Petruzzelli S, Papi A. Extrafine triple therapy delays COPD clinically important deterioration vs ICS/LABA, LAMA, or LABA/LAMA. Int J Chron Obstruct Pulmon Dis. 2019; 14:531-46. | No interesting outcomes |
| Bogart MR, Hopson SD, Shih HC, Stanford RH, Coutinho AD. COPD exacerbation costs in the IMPACT study: a within-trial analysis. Am J Manag Care. 2020; 26:e150-e4. | No interesting outcomes |
| Day NC, Kumar S, Criner G, Dransfield M, Halpin DMG, Han MK, et al. Single-inhaler triple therapy fluticasone furoate/umeclidinium/vilanterol versus fluticasone furoate/vilanterol and umeclidinium/vilanterol in patients with COPD: results on cardiovascular safety from the IMPACT trial. Respir Res. 2020; 21:139. | Sub-analysis |
| Halpin DMG, Dransfield MT, Han MK, Jones CE, Kilbride S, Lange P, et al. The effect of exacerbation history on outcomes in the IMPACT trial. Eur Respir J. 2020; 55. | Sub-analysis |
| Han MK, Criner GJ, Dransfield MT, Halpin DMG, Jones CE, Kilbride S, et al. The Effect of ICS Withdrawal and Baseline Inhaled Treatment on Exacerbations in the IMPACT Study: A Randomized, Double-blind Multicenter Trial. Am J Respir Crit Care Med. 2020; 25:25. | Sub-analysis |
| Lipson DA, Crim C, Criner GJ, Day NC, Dransfield MT, Halpin DMG, et al. Reduction in All-Cause Mortality with Fluticasone Furoate/Umeclidinium/Vilanterol in COPD Patients. Am J Respir Crit Care Med. 2020; 12:12. | Sub-analysis |
| Wang C, Yang T, Kang J, Chen R, Zhao L, He H, et al. Efficacy and Safety of Budesonide/Glycopyrrolate/Formoterol Fumarate Metered Dose Inhaler in Chinese Patients with COPD: A Subgroup Analysis of KRONOS. Adv Ther. 2020; 37:1591-607. | Sub-analysis |

**Table S2**Characteristics of included studies for the analysis of each outcome.

| study | Treatment　(µg) | Duration  (weeks) | Country (N) | Prior  ICS (%) | Primary  outcome | Secondary  outcome | Ex | SG  RQ | TDI | FEV_1_ | AE |
| --- | --- | --- | --- | --- | --- | --- | --- | --- | --- | --- | --- |
| ICS add-on protocol | |  |  |  |  | |  |  |  |  |  |
| Aaron　2007  OPTIMAL | Fluticasone 1000  Tiotoropium 18  Salmeterol 100  (Separate inhalers)  vs  Tiotoropium 18  Salmeterol 100 | 52 | 1 | 76 | Exacerbations | Symptoms,  Lung function |  |  | ✔ |  | ✔ |
|  |  |  |  |  |  | |  |  |  |  |  |
| Ferguson 2018  KRONOS  NCT02497001 | Budesonide 640  Glycopyrronium 36  Formoterol 19.2  (Fixed inhaler)  vs  Glycopyrronium 36  Formoterol 19.2  (Fixed inhaler) | 24 | 4 | 72 | Lung function | Exacerbations,  Symptoms | ✔ | ✔ | ✔ | ✔ |  |
|  |  |  |  |  |  |  |  |  |  |  |  |
| Lipson 2018  IMPACT  NCT02164513 | Fluticasone 100  Umeclidinium　62.5  Vilanterol　25  (Fixed inhaler)  vs  Umeclidinium　62.5  Vilanterol　25  (Fixed inhaler) | 52 | 37 | 72 | Exacerbations | Lung function, Symptoms, Adverse events | ✔ | ✔ |  | ✔ | ✔ |
|  |  |  |  |  |  |  |  |  |  |  |  |
| Papi　2018  TRIBUTE  NCT02579850 | Budesonide 174  Glycopyrronium 18  Formoterol 10  (Fixed inhaler)  vs  Glycopyrronium 43  Indacaterol 85  (Fixed inhaler) | 52 | 17 | 65 | Exacerbations | Lung function | ✔ | ✔ |  |  | ✔ |
|  |  |  |  |  |  |  |  |  |  |  |  |
| Kerwin　2019  Extension study  NCT02536508 | Same as KRONOS | 52 | 4 | 72 | Adverse events |  |  |  |  |  | ✔ |
|  |  |  |  |  |  |  |  |  |  |  |  |
| Rabe　2020  ETHOS NCT02465567 | Budesonide 640  Glycopyrronium 36  Formoterol 19.2  (Fixed inhaler)  vs  Glycopyrronium 36  Formoterol 19.2  (Fixed inhaler) | 52  （TDI 24） | 26 | 80 | Exacerbations | Symptoms, Adverse events | ✔ | ✔ | ✔ |  | ✔ |
|  |  |  |  |  |  |  |  |  |  |  |  |
| Definition of abbreviations: Ex = exacerbations; FEV_1_ = forced expiratory volume in 1 second; AE = adverse events;  ICS = inhaled corticosteroid ; ✔ = included studies. | | | | | | | | | | | |

**Table S2** Characteristics of included studies for the analysis of each outcome (continued)

| study | Treatment　(µg) | Duration  (weeks) | Country (N) | Prior  ICS (%) | Primary  outcome | Secondary  outcome | Ex | SG  RQ | TDI | FEV_1_ | AE |
| --- | --- | --- | --- | --- | --- | --- | --- | --- | --- | --- | --- |
| ICS withdrawal protocol | |  |  |  |  | |  |  |  |  |  |
| Magnussen 2014  WISDOM NCT00975195 | Fluticasone 1000  Tiotoropium 18  Salmeterol 100  (Separate inhalers)  vs  Tiotoropium 18  Salmeterol 100  (Separate inhalers) | 52 | 23 | 70 | Exacerbations | Lung function, Symptoms | ✔ | ✔ |  | ✔ | ✔ |
|  |  |  |  |  |  | |  |  |  |  |  |
| Chapman 2018  SUNSET NCT02603393 | Fluticasone 1000  Tiotoropium 18  Salmeterol 100  (Separate inhalers)  vs  Glycopyrronium 50  Indacaterol 110  (Fixed inhaler) | 26 | 21 | 100 | Lung function | Exacerbations,  Symptoms, Adverse events | ✔ | ✔ | ✔ | ✔ | ✔ |
|  |  |  |  |  |  |  |  |  |  |  |  |
| Definition of abbreviations: Ex = exacerbations; FEV_1_ = forced expiratory volume in 1 second; AE = adverse events;  ICS = inhaled corticosteroid ; ✔ = included studies. | | | | | | | | | | | |

**Table S3**Baseline blood eosinophil count and moderate to severe COPD exacerbations in the past 12 months.

| study | Treatment　(µg) | Eos  ≥ 150 | Eos  ≥ 300 | Ex (0) | Ex (1) | Ex (≥2) |
| --- | --- | --- | --- | --- | --- | --- |
| ICS add-on protocol | |  |  |  |  |  |
| Aaron　2007  OPTIMAL | ICS/LAMA/LABA  vs  LAMA/LABA | NA  NA | NA  NA | NA  NA | NA  NA | NA  NA |
|  |  |  |  |  |  |  |
| Ferguson 2018  KRONOS  NCT02497001 | ICS/LAMA/LABA  vs  LAMA/LABA | 50.9  53.4 | NA  NA | 73.4  75.7 | 19.6  17.3 | 7.0  7.0 |
|  |  |  |  |  |  |  |
| Lipson 2018  IMPACT  NCT02164513 | ICS/LAMA/LABA  vs  LAMA/LABA | 57  (total data) | NA | <1  <1 | 45  45 | 55  55 |
|  |  |  |  |  |  |  |
| Papi　2018  TRIBUTE  NCT02579850 | ICS/LAMA/LABA  vs  LAMA/LABA | 240*  230* | | 0  0 | 80  82 | 20  18 |
|  |  |  |  |  |  |  |
| Kerwin　2019  Extension study  NCT02536508 | ICS/LAMA/LABA  vs  LAMA/LABA | 64.9  69.0 | NA  NA | 78.4  74.1 | 17.0  19.5 | 4.6  6.3 |
|  |  |  |  |  |  |  |
| Rabe　2020  ETHOS NCT02465567 | ICS/LAMA/LABA  vs  LAMA/LABA | 59.8  60.0 | 14.5  13.8 | 0.1  0.1 | 44.0  42.8 | 55.9  57.1 |
| ICS withdrawal protocol | |  |  |  |  |  |
| Magnussen 2014  WISDOM NCT00975195 | ICS/LAMA/LABA  vs  LAMA/LABA | 51.0  (total data) | 19.5  (total data) | NA  NA | NA  NA | NA  NA |
|  |  |  |  |  |  |  |
| Chapman 2018  SUNSET NCT02603393 | ICS/LAMA/LABA  vs  LAMA/LABA | NA  NA | 22.7  23.8 | 68.4  63.4 | 31.6  36.6 | 0  0 |
|  |  |  |  |  |  |  |
| ICS = inhaled corticosteroid; LAMA = long acting muscarinic receptor antagonist; LABA = long acting β_2_ adrenoreceptor agonist; Eos = eosinophil; data are shown as percentage of participants with ≥ 150 or ≥ 300 (cells/mm^3^) blood eosinophil count, (%); * data are shown as mean eosinophil cell count, (cells/mm^3^); Ex = exacerbations; data are shown as percentage of participants with 0, 1 or ≥2 moderate to severe COPD exacerbations in the past 12 months, (%) ; NA = not available. | | | | | | |

**Table S4** Assessment of risk of bias.

**ICS add-on protocol**


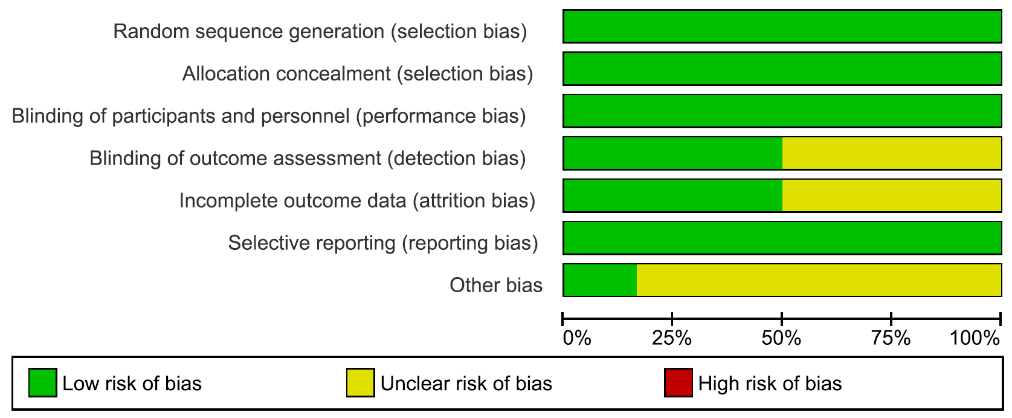


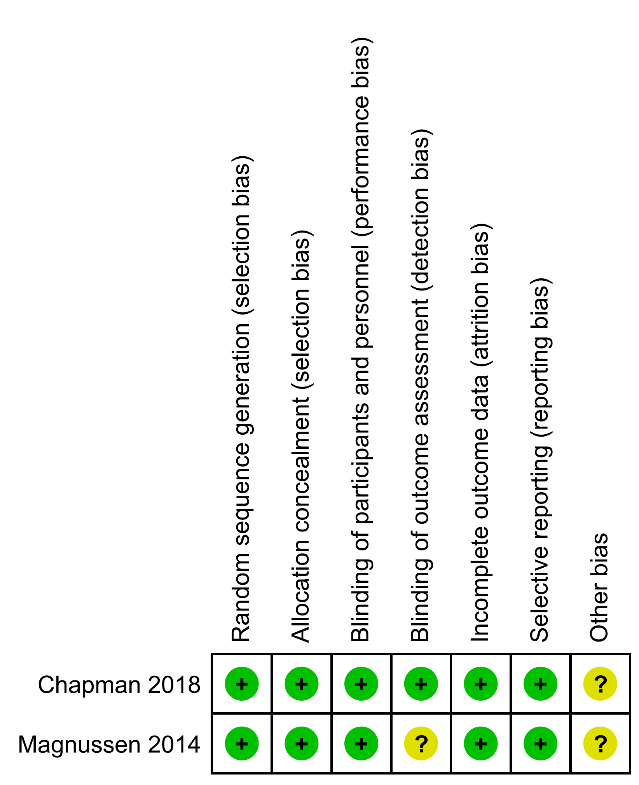

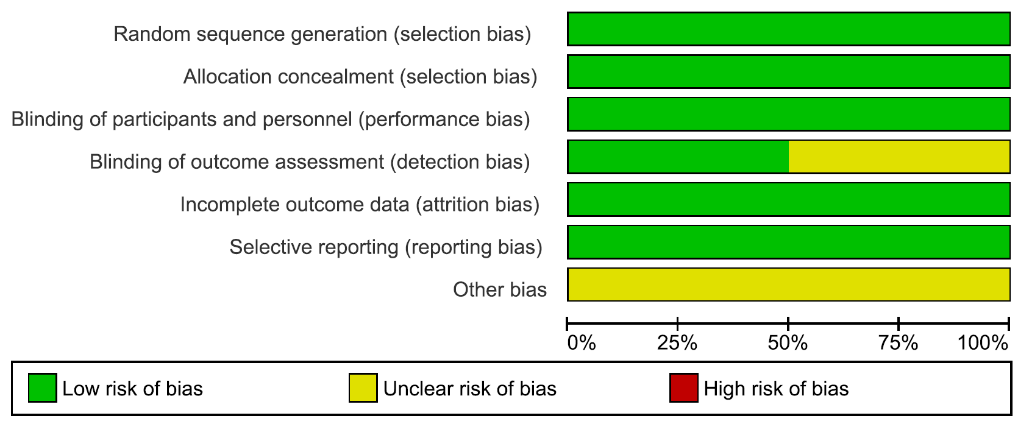


**ICS withdrawal protocol**

**Table S5** Details for the risk of bias assessment.

**Table S6** Sub-analysis of exacerbations by baseline blood eosinophil count (descriptive analysis).

| study | Treatment　(µg) | Eos  < 150 | Eos  ≥ 150 | Eos  < 300 | Eos  ≥ 300 |
| --- | --- | --- | --- | --- | --- |
| ICS add-on protocol | |  |  |  |  |
|  |  |  |  |  |  |
| Ferguson 2018  KRONOS  NCT02497001 | ICS/LAMA/LABA  vs  LAMA/LABA | 0.61  (0·41–0.90) | 0.39  (0·27–0.58) | NA | NA |
|  |  |  |  |  |  |
| Lipson 2018  IMPACT  NCT02164513 | ICS/LAMA/LABA  vs  LAMA/LABA | Annual rate  0.85  (0.80 to 0.91)  0.97  (0.88 to 1.07) | Annual rate  0.95  (0.90 to 1.01)  1.39  (1.29 to 1.51) | NA | NA |
|  |  |  |  |  |  |
| Papi　2018  TRIBUTE  NCT02579850 | ICS/LAMA/LABA  vs  LAMA/LABA | Eos <　2%  0·94  (0·71–1·25; p=0·685) | Eos ≥　2%  0·81  (0·66–0·98; p=0·029) | Eos <　200  0·87  (0·69–1·10； p=0·244) | Eos ≥　200  0·81  (0·65–1·01; p=0·057) |
|  |  |  |  |  |  |
| Rabe　2020  ETHOS NCT02465567 | ICS/LAMA/LABA  vs  LAMA/LABA | Hazard ratio  0·87  (0·75–1·02) | Hazard ratio  0·68  (0·61–0.77) | NA | NA |
| ICS withdrawal protocol | |  |  |  |  |
| Chapman 2018  SUNSET NCT02603393 | ICS/LAMA/LABA  vs  LAMA/LABA | NA | NA | 0.97  (0.72 to 1.32) | 1.86  (1.06 to 3.29) |
|  |  |  |  |  |  |
| ICS = inhaled corticosteroid; LAMA = long acting muscarinic receptor antagonist; LABA = long acting β_2_ adrenoreceptor agonist; Eos = eosinophil; NA = not available; data are shown as rate ratio (95%CI) in the incidence of moderate or severe COPD exacerbations of participants with < 150, ≥ 150, < 300 or ≥ 300 (cells/mm^3^) baseline blood eosinophil counts between ICS/LAMA/LABA and LAMA/LABA treatment group; in the ICS withdrawal protocol, rate ratio is shown as the incidence of exacerbations in LAMA/LABA treatment per that in ICS/LAMA/LABA treatment. | | | | | |

**Table S7** Sub-analysis of trough FEV_1_ by baseline blood eosinophil count (descriptive analysis).

| study | Treatment　(µg) | Eos  < 150 | Eos  ≥ 150 | Eos  < 300 | Eos  ≥ 300 |
| --- | --- | --- | --- | --- | --- |
| ICS add-on protocol | |  |  |  |  |
| Ferguson 2018  KRONOS  NCT02497001 | ICS/LAMA/LABA  vs  LAMA/LABA | 0.002  (-0.02 to 0.024) | 0.038  (0.011 to 0.065) | NA | NA |
| ICS withdrawal protocol | |  |  |  |  |
| Chapman 2018  SUNSET NCT02603393 | ICS/LAMA/LABA  vs  LAMA/LABA | NA | NA | -0.013  (-0.044 to 0.017) | -0.069  (-0.125 to -0.012) |
|  |  |  |  |  |  |
| Definition of abbreviations: ICS = inhaled corticosteroid; LAMA = long acting muscarinic receptor antagonist; LABA = long acting β_2_ adrenoreceptor agonist; Eos = eosinophil; NA = not available. Data are shown as mean difference (L) (95%CI) in the trough FEV_1_ of participants with < 150, ≥ 150, < 300 or ≥ 300 (cells/mm^3^) baseline blood eosinophil counts between ICS/LAMA/LABA and LAMA/LABA treatment group. In the ICS withdrawal protocol, mean difference is shown as the diffference of trough FEV_1_ in LAMA/LABA treatment compared with that in ICS/LAMA/LABA treatment. | | | | | |

**Table S8** Summary of findings for the main comparison.

**Figure S1** Comparison of exacerbation rate in each trial.

**Figure S2** Efficacy of ICS add-on to LAMA/LABA on total adverse events.

**Figure S3** Efficacy of ICS add-on to LAMA/LABA on serious adverse events.

**
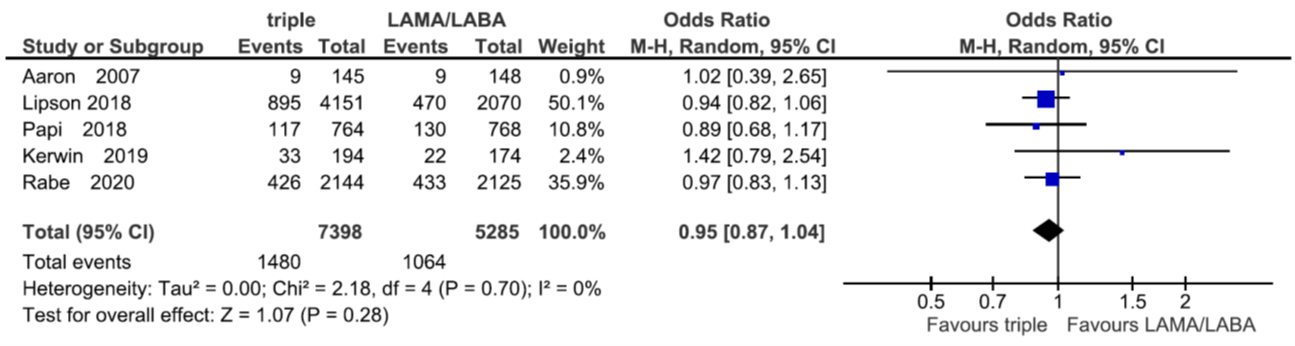
**

**Figure S4** Sub-analysis of cause of mortality: cardiovascular events.

**
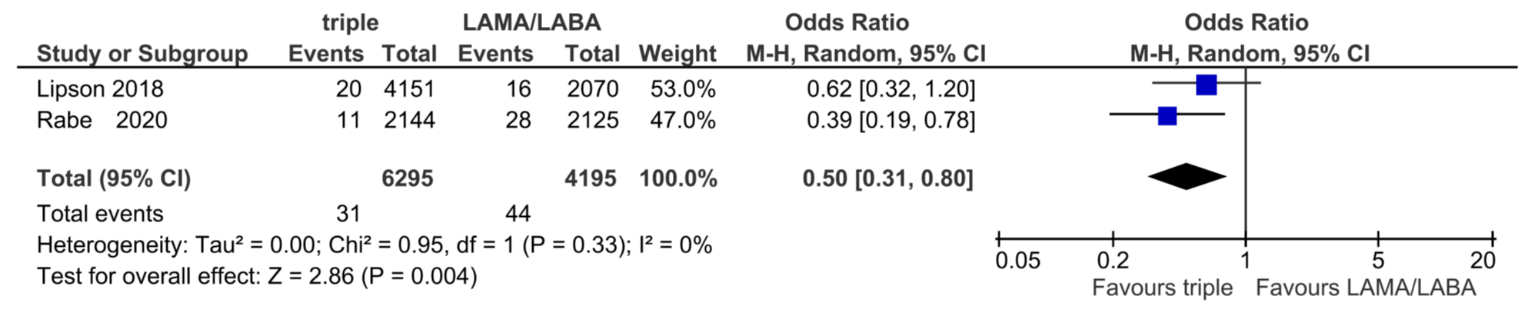
**

**Figure S5** Sub-analysis of exacerbations by history of exacerbations and CAT score.

**
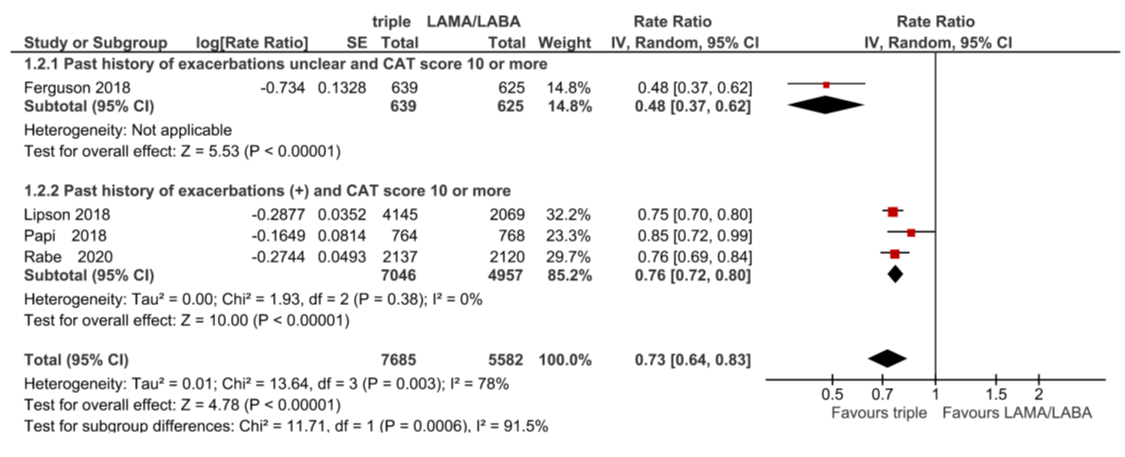
**

**Figure S6** Sub-analysis of SGRQ score by history of exacerbations and CAT score.

**
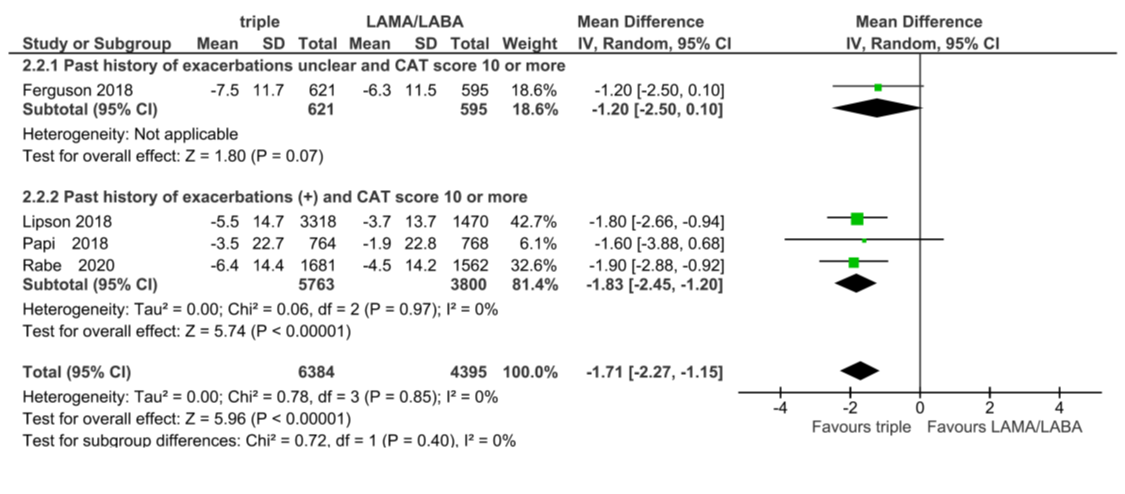
Figure S7** Sub-analysis of TDI sore by history of exacerbations and CAT score.

**_
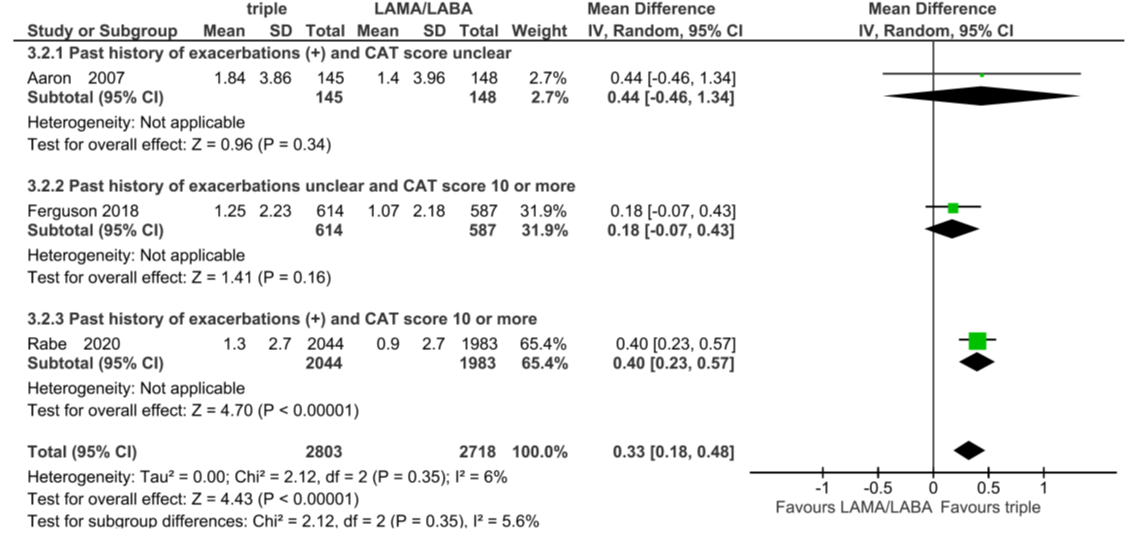
_**

**Figure S8** Sub-analysis of trough FEV_1_ by history of exacerbations and CAT score.

**
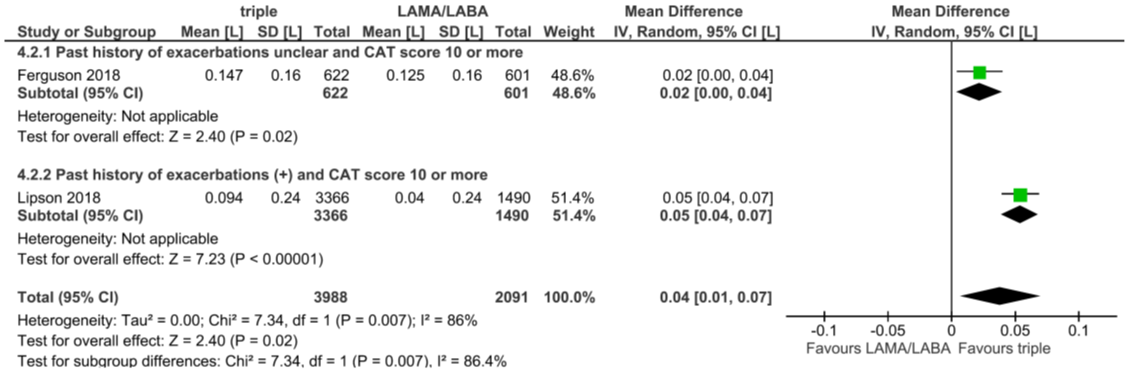
****Figure S9** Sub-analysis of pneumonia events by history of exacerbations and CAT score.

**
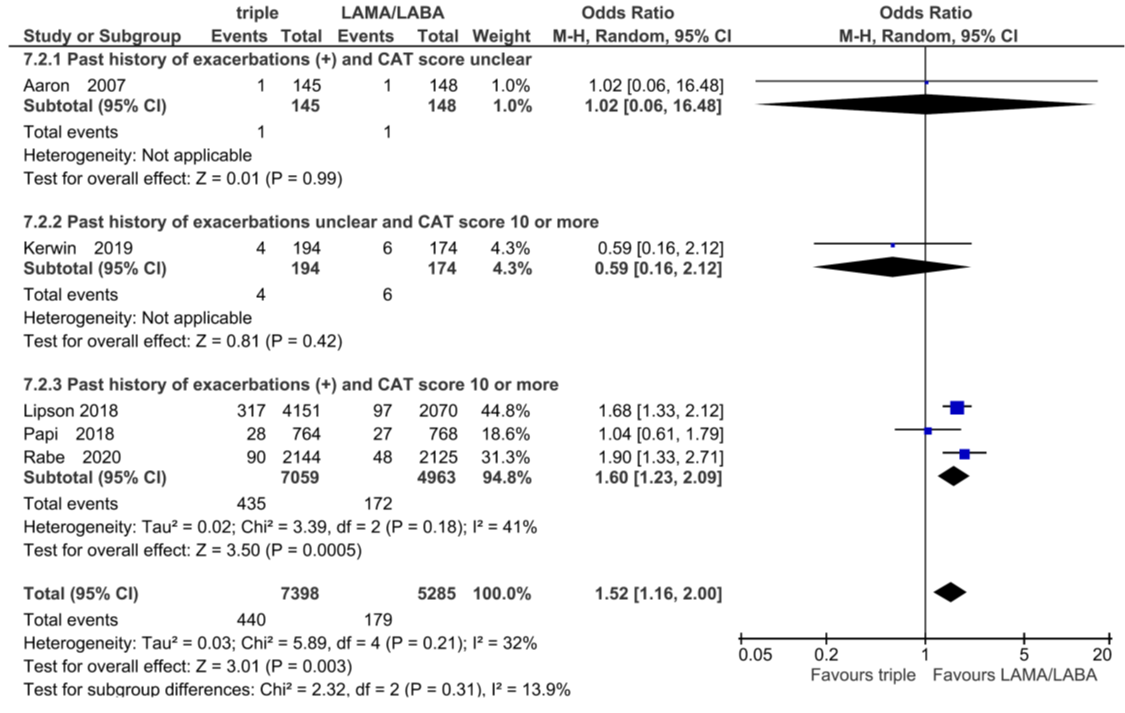
**

**Figure S10** Sub-analysis of mortality rate by history of exacerbations and CAT score.

**
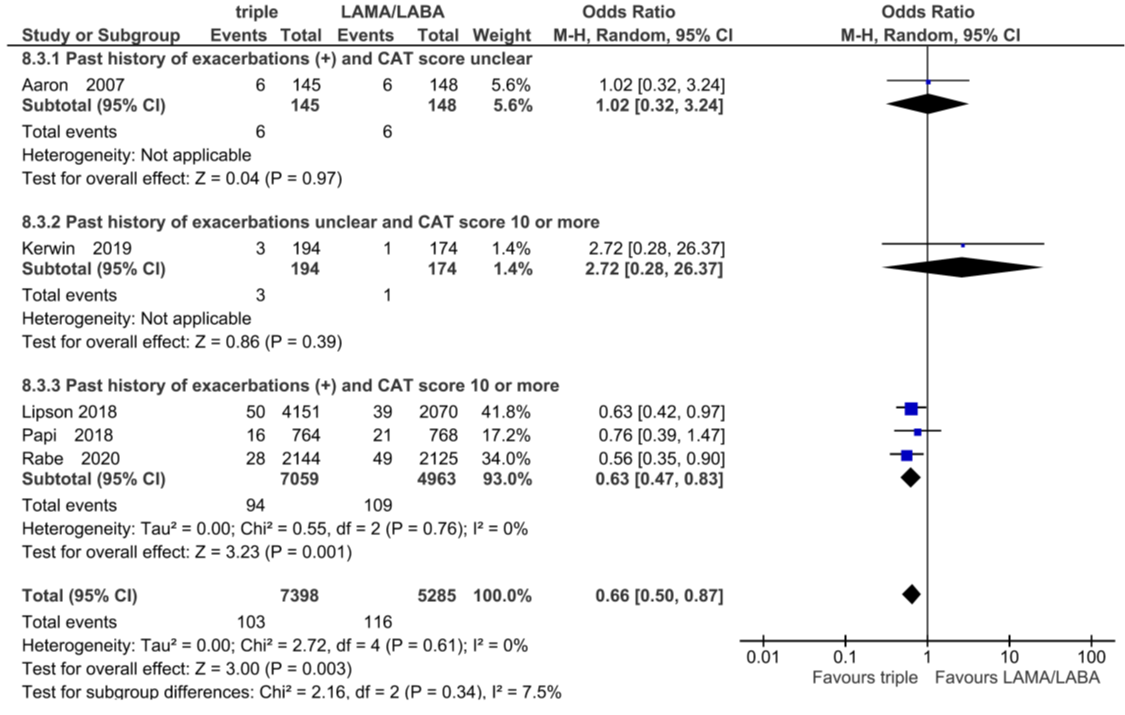
**

**Figure S11** Efficacy of ICS withdrawal from ICS/LAMA/LABA on exacerbations.

Rate ratio shown as the incidence of exacerbations in LAMA/LABA treatment per that in ICS/LAMA/LABA treatment.

**
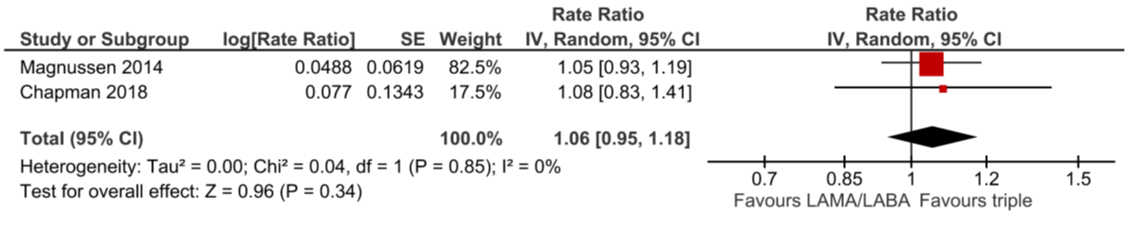
**

**Figure S12** Comparison between ICS add-on and ICS withdrawal protocol: change from baseline in SGRQ score.

**
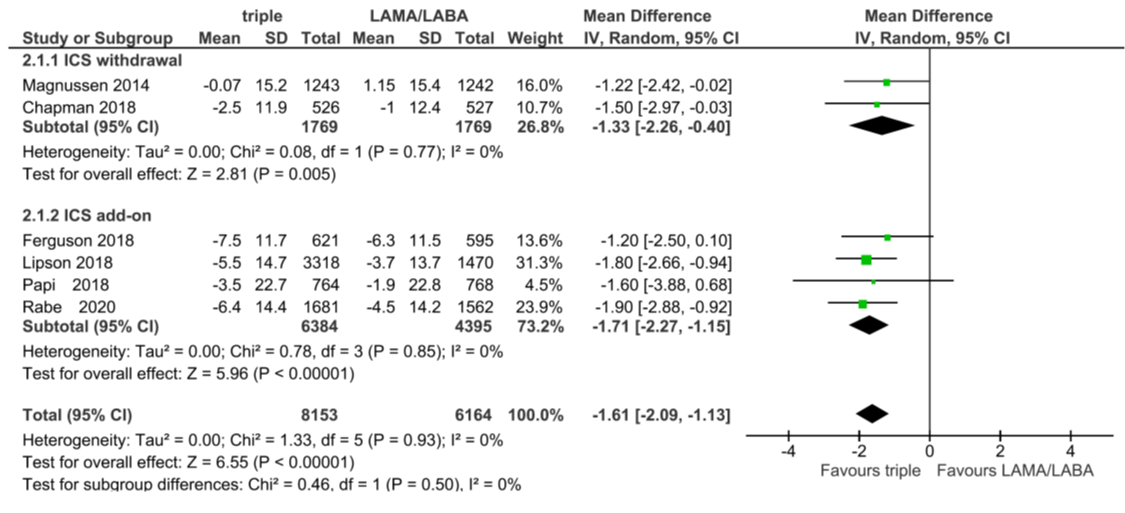
**

**Figure S13** Comparison between ICS add-on and ICS withdrawal protocol: change from baseline in TDI score.

**
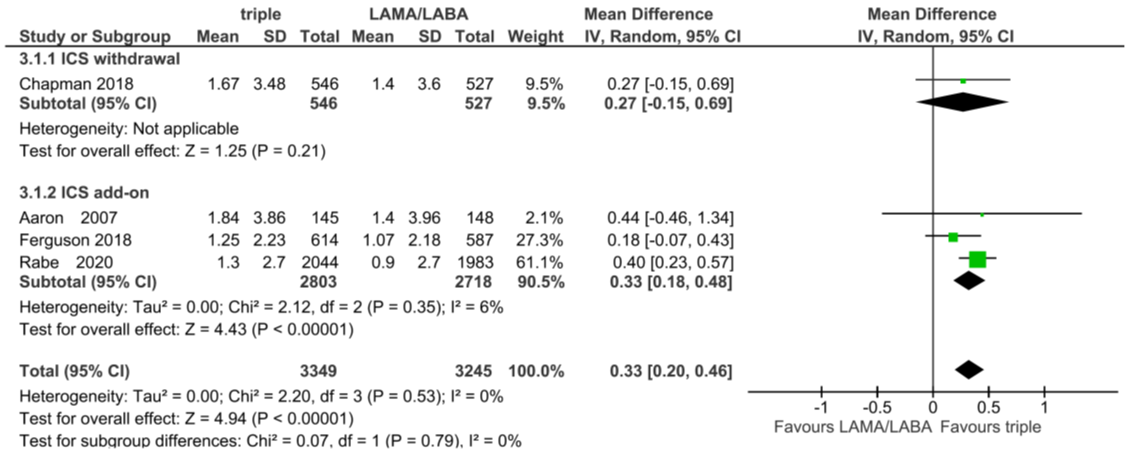
**

**Figure S14** Comparison between ICS add-on and ICS withdrawal protocol: trough FEV_1_.

**
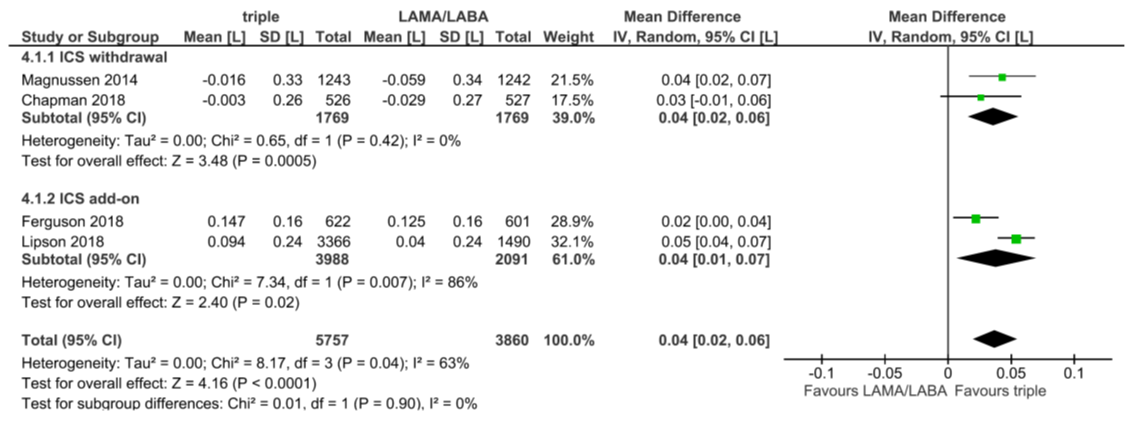
**

**Figure S15** Comparison between ICS add-on and ICS withdrawal protocol: total adverse events.

**
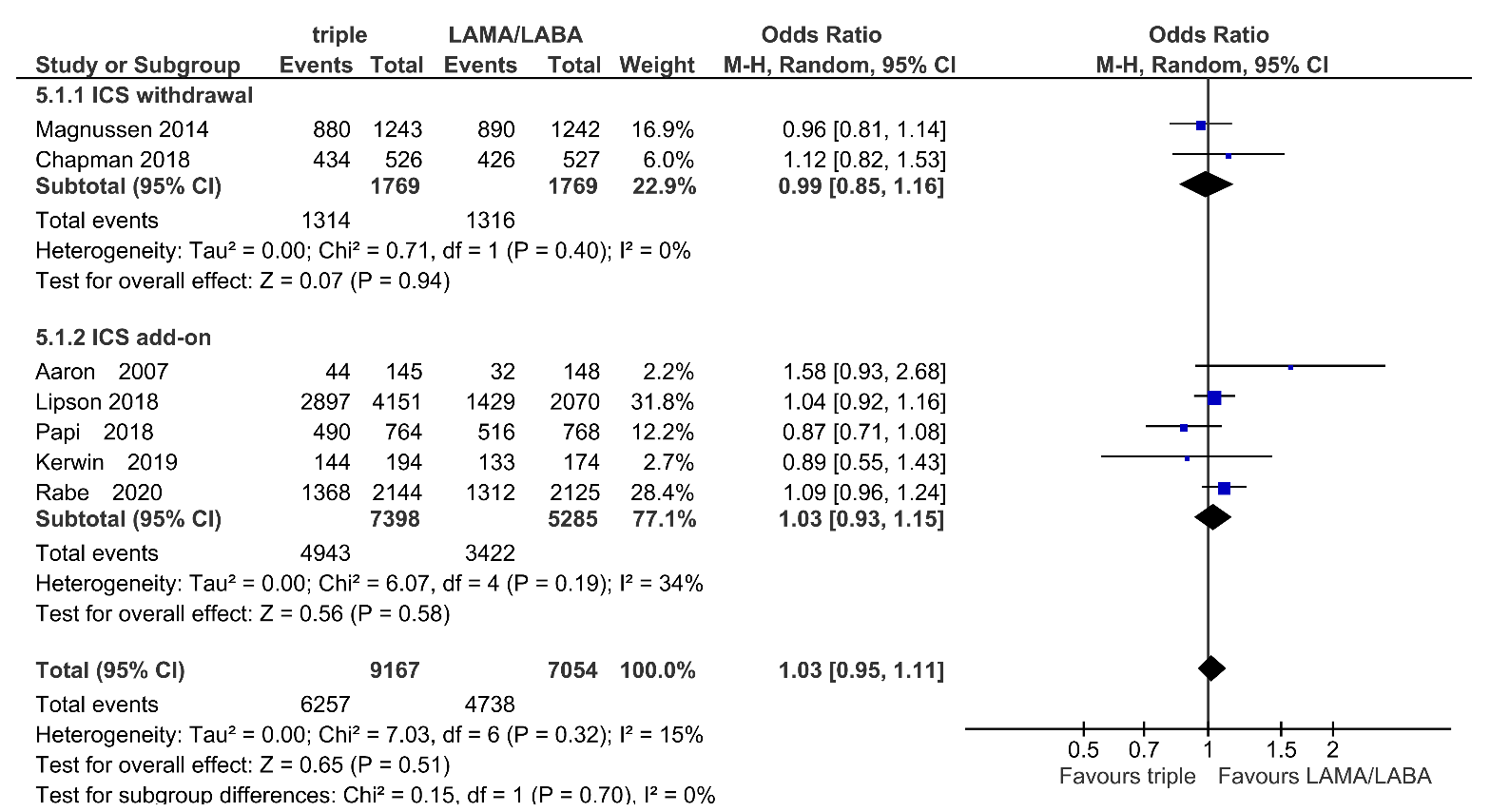
**

**Figure S16** Comparison between ICS add-on and ICS withdrawal protocol: serious adverse events.

**
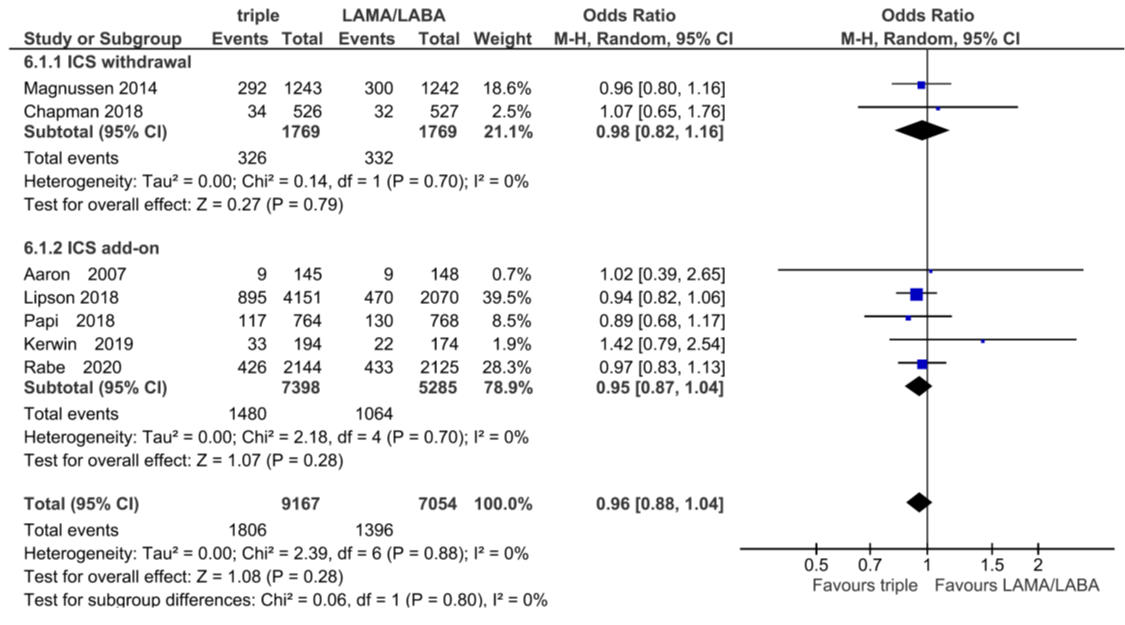
**

**Figure S17** Comparison between ICS add-on and ICS withdrawal protocol: pneumonia events.

**
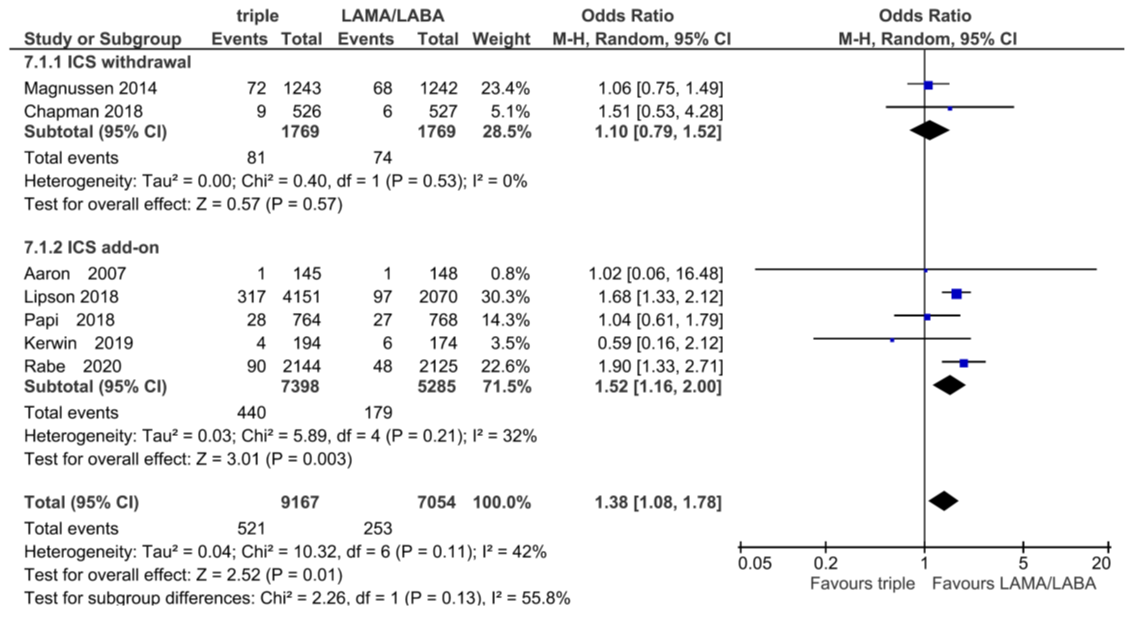
**

**Figure S18** Comparison between ICS add-on and ICS withdrawal protocol: mortality.

**
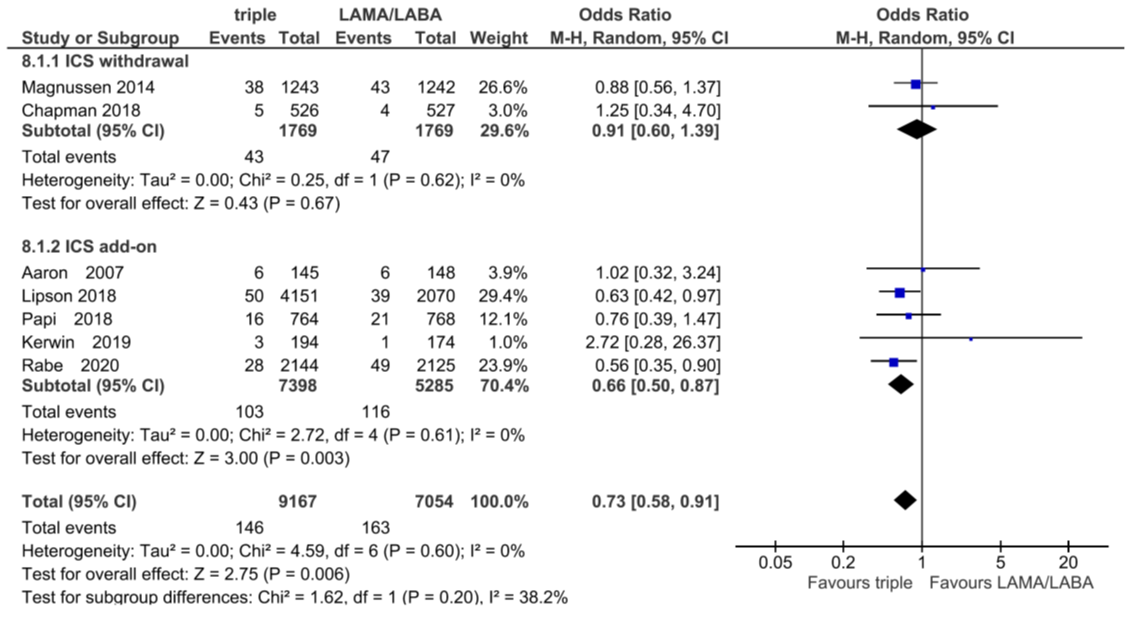
**
